# GEOexplorer: a webserver for gene expression analysis and visualisation

**DOI:** 10.1093/nar/gkac364

**Published:** 2022-05-24

**Authors:** Guy P Hunt, Luigi Grassi, Rafael Henkin, Fabrizio Smeraldi, Thomas P Spargo, Renata Kabiljo, Sulev Koks, Zina Ibrahim, Richard J B Dobson, Ammar Al-Chalabi, Michael R Barnes, Alfredo Iacoangeli

**Affiliations:** Department of Biostatistics and Health Informatics, Institute of Psychiatry, Psychology and Neuroscience, King's College London, London, UK; Department of Basic and Clinical Neuroscience, Maurice Wohl Clinical Neuroscience Institute, Institute of Psychiatry, Psychology and Neuroscience, King's College London, London, SE5 9NU, UK; Perron Institute for Neurological and Translational Science, Nedlands, WA 6009, Australia; Centre for Molecular Medicine and Innovative Therapeutics, Murdoch University, Murdoch, WA 6150, Australia; Biopharmaceutical Development, BioPharmaceuticals R&D, AstraZeneca, Cambridge, CB21 6GH, UK; Centre for Translational Bioinformatics, William Harvey Research Institute, Faculty of Medicine and Dentistry, Queen Mary University of London, Charterhouse Square, London, EC1M 6BQ, UK; School of Electronic Engineering and Computer Science, Queen Mary University of London, Mile End Road, London E1 4NS, UK; Department of Basic and Clinical Neuroscience, Maurice Wohl Clinical Neuroscience Institute, Institute of Psychiatry, Psychology and Neuroscience, King's College London, London, SE5 9NU, UK; Department of Biostatistics and Health Informatics, Institute of Psychiatry, Psychology and Neuroscience, King's College London, London, UK; Perron Institute for Neurological and Translational Science, Nedlands, WA 6009, Australia; Centre for Molecular Medicine and Innovative Therapeutics, Murdoch University, Murdoch, WA 6150, Australia; Department of Biostatistics and Health Informatics, Institute of Psychiatry, Psychology and Neuroscience, King's College London, London, UK; Department of Biostatistics and Health Informatics, Institute of Psychiatry, Psychology and Neuroscience, King's College London, London, UK; Institute of Health Informatics, University College London, London, UK; National Institute for Health Research Biomedical Research Centre and Dementia Unit at South London and Maudsley NHS Foundation Trust and King's College London, London, UK; Department of Basic and Clinical Neuroscience, Maurice Wohl Clinical Neuroscience Institute, Institute of Psychiatry, Psychology and Neuroscience, King's College London, London, SE5 9NU, UK; King′s College Hospital, Bessemer Road, Denmark Hill, London, SE5 9RS, UK; Centre for Translational Bioinformatics, William Harvey Research Institute, Faculty of Medicine and Dentistry, Queen Mary University of London, Charterhouse Square, London, EC1M 6BQ, UK; The Alan Turing Institute, London NW1 2DB, UK; Department of Biostatistics and Health Informatics, Institute of Psychiatry, Psychology and Neuroscience, King's College London, London, UK; Department of Basic and Clinical Neuroscience, Maurice Wohl Clinical Neuroscience Institute, Institute of Psychiatry, Psychology and Neuroscience, King's College London, London, SE5 9NU, UK; National Institute for Health Research Biomedical Research Centre and Dementia Unit at South London and Maudsley NHS Foundation Trust and King's College London, London, UK

## Abstract

Gene Expression Omnibus (GEO) is a database repository hosting a substantial proportion of publicly available high throughput gene expression data. Gene expression analysis is a powerful tool to gain insight into the mechanisms and processes underlying the biological and phenotypic differences between sample groups. Despite the wide availability of gene expression datasets, their access, analysis, and integration are not trivial and require specific expertise and programming proficiency. We developed the GEOexplorer webserver to allow scientists to access, integrate and analyse gene expression datasets without requiring programming proficiency. Via its user-friendly graphic interface, users can easily apply GEOexplorer to perform interactive and reproducible gene expression analysis of microarray and RNA-seq datasets, while producing a wealth of interactive visualisations to facilitate data exploration and interpretation, and generating a range of publication ready figures. The webserver allows users to search and retrieve datasets from GEO as well as to upload user-generated data and combine and harmonise two datasets to perform joint analyses. GEOexplorer, available at https://geoexplorer.rosalind.kcl.ac.uk, provides a solution for performing interactive and reproducible analyses of microarray and RNA-seq gene expression data, empowering life scientists to perform exploratory data analysis and differential gene expression analysis on-the-fly without informatics proficiency.

## Graphical Abstract

**Graphical Abstract:**
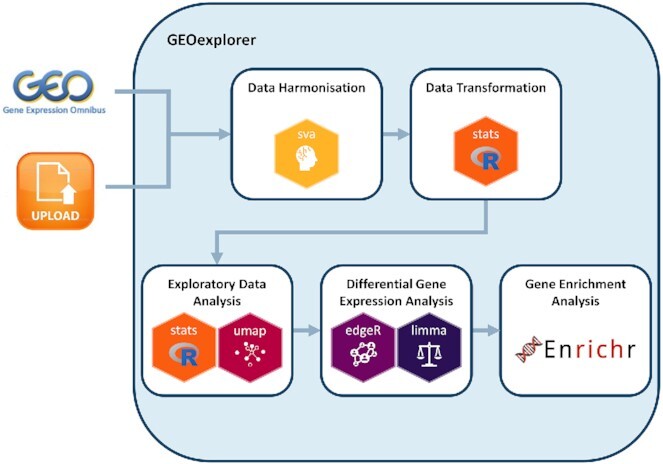
GEOexplorer (https://geoexplorer.rosalind.kcl.ac.uk) is a webserver for exploratory data analysis, differential gene expression analysis and gene enrichment analysis of GEO and user uploaded gene expression datasets from RNA-seq and microarray experiments.

## INTRODUCTION

The analysis of gene expression is a powerful tool to investigate the molecular basis of phenotypic and biological differences across groups of biological samples (1). For example, comparing gene expression levels in cells affected by disease versus their unaffected counterparts might highlight key pathways involved in disease development or pathogenesis. Over the past three decades, there have been continuous advances in the techniques for the generation of high-throughput gene expression data that have led to rapid growth in the number of gene expression studies being performed. A substantial proportion of these studies publish their datasets in the Gene Expression Omnibus (GEO), which is a public repository that makes gene expression datasets freely available (2).

To date, expression data for 170,000 series with almost 5,000,000 samples have been deposited in GEO. Despite this unprecedented availability of high quality and freely accessible data, analysing them can be difficult. This is due to several factors including the high dimensionality of the datasets, variation in experimental structures including the platform and technology used, and the statistical, programming and bioinformatic proficiency required to perform the analyses. End-to-end protocols for gene expression analysis exist (3–5) and several R packages have been developed that collectively provide researchers with a comprehensive analysis framework (6–8). However, their use requires reasonable programming ability. To overcome this, the GEO2R tool was developed. It is available at, https://www.ncbi.nlm.nih.gov/geo/geo2r/, enabling users to perform gene expression analysis on individual GEO microarray datasets without requiring significant programming skills (2). Even if GEO2R substantially meets the need for an intuitive and accessible interface to perform gene expression analysis on microarray datasets hosted on GEO, it is limited to a single dataset at a time and, does not allow for the processing of the RNA-seq datasets hosted on GEO or of other external datasets.

To improve the usability of the available gene expression datasets, we have developed GEOexplorer, a user-friendly webserver for on-the-fly analysis of gene expression data. GEOexplorer enables users with no programming skills to browse and retrieve RNA-seq and microarray expression datasets from GEO, or to upload their own data, to perform an end-to-end gene expression analysis. The webserver provides a rich selection of analytical techniques, enables in-depth exploration of the gene expression analysis results using interactive visualisations, and generates publication ready figures. Moreover, GEOexplorer allows the combining and harmonising of two datasets interactively to perform powerful integrated analyses.

GEOexplorer takes as input either expression data in CSV format, or a GEO series ID, also known as a GEO accession code, which is used to extract the gene expression dataset and experimental information from GEO. The GEO series IDs can be identified on the webserver using the GEO search utility that allows searches for GEO datasets using keywords. Gene expression analysis usually occurs in two stages. The first stage is called ‘Exploratory Data Analysis’ (EDA) which is used to gain an overall understanding of the gene expression dataset. The second stage is called ‘Differential Gene Expression Analysis’ (DGEA) which identifies the probes (or the genes/transcripts in case of an RNA-seq experiment) that are differentially expressed between two groups. In addition to EDA and DGEA, GEOexplorer performs ‘Gene Enrichment Analysis’ (GEA) to incorporate the biological context of differentially expressed genes. GEOexplorer makes performing EDA, DGEA and GEA easy by requiring minimal manual configurations, allowing the analysis to be automated, and it adheres to the FAIR Guiding Principles for scientific data (9). GEOexplorer is publicly available without requiring user registration at https://geoexplorer.rosalind.kcl.ac.uk and as an R package at http://bioconductor.org/packages/release/bioc/html/GEOexplorer.html (latest release) and at https://github.com/KHP-Informatics/GEOexplorer (development version).

## MATERIALS AND METHODS

### Implementation

GEOexplorer is written in the R programming language and relies on the functionality of several widely used packages available from Bioconductor and CRAN. The analysis of microarray datasets largely follows the workflow and packages outlined in GEO2R (2) whilst the analysis of RNA-seq datasets largely follows the workflow and packages outlined by Law et al. (5). In addition to these workflows, several other R packages are used to perform additional analyses which are discussed in the workflow section.

The GEOexplorer webserver was developed using the Shiny R framework, available from https://cran.r-project.org/web/packages/shiny/index.html. The layout of the user interface is built with a sidebar containing widgets for the data collection and transformation options. The main panel is structured with four different tabs that mirror the different steps to perform gene expression analysis. The steps include reviewing the dataset information, the results of EDA, the results of DGEA and the results of GEA. The widgets for DGEA and GEA options are available within dedicated tabs. Each tab has multiple sub-tabs to guide the user through the results.

The *plotly* (10) graphics system is used to generate interactive visualisations, enabling interactions by brushing or clicking on them in the Shiny framework. Interactive heatmaps are generated with the *heatmaply* R package (11), and tables are displayed as interactive objects for efficient navigation via the *DT* R package, available from https://cran.r-project.org/web/packages/DT/index.html.

The functionality of GEOexplorer is extensively described in the ‘about’, ‘workflow’ and ‘tutorial’ tabs in the webserver navigation bar.

### Workflow overview

**Figure 1.**
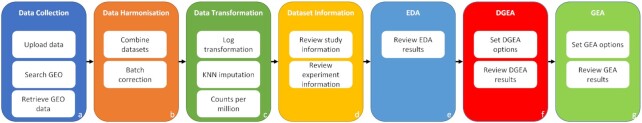
GEOexplorer workflow overview. **(A)** GEOexplorer's workflow begins with the users selecting the data source of their gene expression dataset, either GEO or user upload. GEOexplorer will automatically source GEO microarray datasets and several formats of GEO RNA-seq datasets. Users can also upload their own gene expression datasets. GEOexplorer enables users to search for GEO datasets. (**B)** Users can select to combine two gene expression datasets and then perform batch correction, so they are comparable. (**C)** Log_2_ transformation and k-nearest neighbour (KNN) imputation can be selected before analysing microarray data. Log_2_ and counts per million transformations can be selected before analysing RNA-seq data. (**D)** Dataset details, including information about the study and experiment, can be reviewed. (**E)** Results of EDA can be reviewed. (**F)** Options for DGEA can be set based on the outputs of EDA. Subsequently, the outputs of DGEA can be reviewed. (**G)** Options for GEA can be set. Subsequently, the outputs of GEA can be reviewed.

The user can explore datasets available in GEO using the relevant GEO series IDs or upload their own expression data in CSV format. One or two datasets can be analysed at the same time, by selecting the ‘single’ or ‘combine’ option in the sidebar. When combining two datasets, batch correction can be performed using the *sva* R package, available from https://bioconductor.org/packages/release/bioc/html/sva.html, empirical Bayes method or the *limma* linear model method (6). The ‘GEO search’ tab, present in the navigation bar, gives the possibility to retrieve the GEO series IDs of available datasets, by querying the GEO database with keywords or phrases such as a paper title or author name. If different platforms are present in a given dataset the user can select the one to use. Moreover, it can be decided whether to log_2_ transform the data or let the server auto-detect the need for this processing (default option). Log_2_ transformation autodetection applies log_2_ transformation if quantile 0.99 is greater than 100 or if quantile 0.25 is greater than 0 and quantile 1 - quantile 0 is greater than 50. KNN imputation can be performed on microarray datasets to fill in missing values using the *impute* R package, available from https://bioconductor.org/packages/release/bioc/html/impute.html, and RNA-seq datasets can be converted to counts per million using the *edgeR* R package (8,12,13). The transformed dataset is subsequently used by GEOexplorer for EDA and several plots are produced to assess the quality and general characteristics of the analysed dataset including the expression density plots, box-and-whisker plot and mean-variance plot. The *stats* R package, available from https://www.r-project.org/, is used to generate the density distributions of each experimental condition, the covariances/correlations between each experimental condition and perform principal component analysis (PCA). Upon the inspection of the EDA results, the user can set the DGEA options, such as whether to apply limma precision weights or force normalisation and run DGEA. Finally, the outputs of DGEA can be reviewed and differentially expressed genes can be used to perform GEA based on Enrichr (14–16) with an extensive collection of phenotypic and biological databases (Figure 1). All figures generated and displayed in the user interface, many of which can be explored interactively on the webserver, are publication-ready quality and can be downloaded with a mouse click in PNG format.

## RESULTS

### Data collection, harmonisation, and transformation

**Figure 2.**
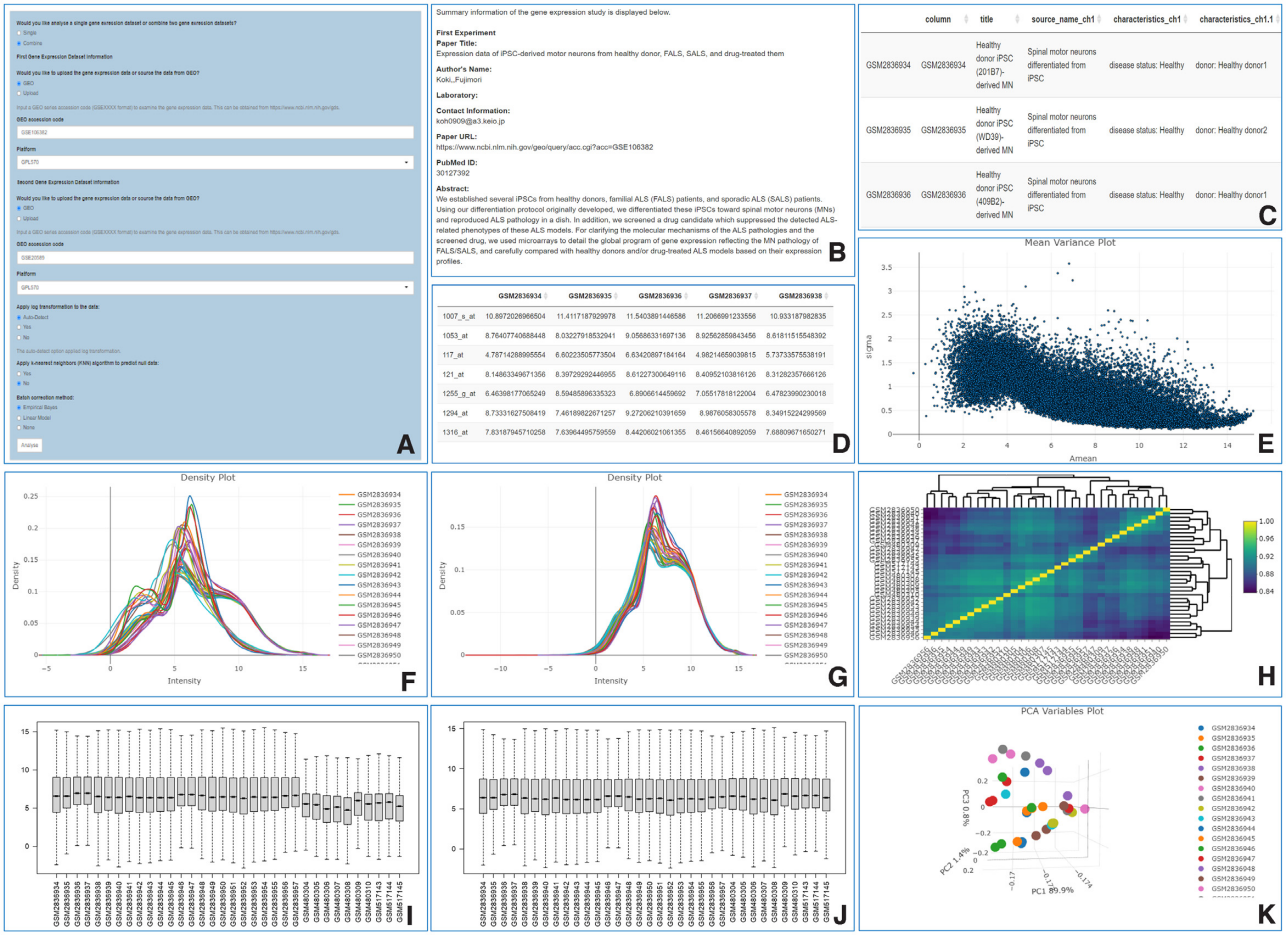
GEOexplorer data collection, harmonisation, and transformation settings, study and experiment information, and EDA outputs. **(A)** GEOexplorer data collection, harmonisation, and transformation settings. (**B)** Experiment information. (**C)** Experimental conditions information. (**D)** Gene expression dataset. (**E)** Mean-variance plot. (**F**) Expression density plot pre-batch correction. (**G)** Expression density plot post-empirical Bayes batch correction. (**H)** Heatmap plot. (**I)** Box-and-whisper plot pre-batch correction. (**J)** Box-and-whisper plot post-empirical Bayes batch correction. (**K)** 3D PCA variables.

To test and demonstrate GEOexplorer's functionalities, we explored, subsampled and combined two microarray GEO datasets, GSE106382 and GSE20589. Both datasets used the same platform, GPL570. The GSE106382 study generated expression data of induced pluripotent stem cells (iPSCs) from healthy controls, sporadic amyotrophic lateral sclerosis (ALS) and familial ALS patients including a subgroup of familial cases who carried a pathogenic mutation in the SOD1 gene (SOD1 ALS). The iPSCs were then differentiated into spinal motor neurons and grown to reproduce ALS pathology (17). In this example, we will focus on the SOD1 ALS samples and therefore we will subsequently refer to these as model SOD1 spinal motor neurons. The GSE20589 study collected cervical spinal motor neurons from healthy controls and SOD1-related ALS post-mortem (18). We used GEOexplorer to combine these two datasets to investigate the differences between the gene expression profiles of the model SOD1 spinal motor neurons and the post-mortem SOD1 spinal motor neurons. Both datasets were automatically sourced by GEOexplorer and transformed as indicated in Figure 2A.

### Reviewing the dataset information

Upon clicking the ‘Analyse’ button in the sidebar, information about the study and experiment is displayed in the ‘Dataset Information’ tab which consists of three subtabs. The transformational settings are also applied to the gene expression dataset. An overview of the study is available in the ‘Experiment Information’ subtab (Figure 2B). This enables the users to validate that they sourced the gene expression dataset(s) from the correct study as well as access information such as the author and abstract. The experiment conditions are listed in the ‘Experimental Conditions Information*’* subtab (Figure 2C). This provides information on the variables tested in the experiment(s). From our example, we can see there are gene expression data available for several healthy controls and ALS spinal motor neurons in both datasets. The gene expression dataset is available in the ‘Gene Expression Dataset’ subtab (Figure 2D).

### Evaluating the results of EDA

Several plots are produced as a result of EDA. For example, GEOexplorer displays an interactive mean-variance plot (Figure 2E). The mean-variance plot displays the log residual standard deviation versus the average log expression of the linear model for each probe (or gene/transcript in case of an RNA-seq experiment). This can be used to assess if there is a lot of variation within the gene expression dataset after fitting it to a linear model (5,6). The level of variation can be used to determine whether to apply the precision weights option during DGEA for microarray datasets. If there is a strong mean-variance trend in the gene expression dataset the precision weights can improve the accuracy of DGEA. In our example, from Figure 2E, we can see there is a mean-variance trend, therefore limma precision weights should be applied during DGEA.

GEOexplorer calculates the density distributions of each experimental condition and displays them in two interactive expression density plots, one which is 2D (Figure 2F, G) and one which is 3D. GEOexplorer displays a box-and-whisker plot (Figure 2I, J) presenting the distribution of probe (or gene/transcript in case of an RNA-seq experiment) expression values for each experimental condition including the min, max, median, 1^st^ quartile and 3^rd^ quartile. The Gene Expression Dataset subtab and the density and box-and-whisker plots are useful for identifying whether the gene expression datasets are normalised or not. For microarray data, if the density plot density curves do not appear to be normally distributed, then it is advisable to force normalisation during DGEA. Equally, if the box-and-whisker plot distributions are not median-centred it indicates the data are not normalised (19) and the user should configure forced normalisation during DGEA. For RNA-seq, the user needs to verify that read counts are not normalised, i.e. integer values in the Gene Expression Dataset subtab, in which case the data can be normalised and taken forward for DGEA. The density and box-and-whisker plots are also useful for visualising batch effects when combining two datasets (Figure 2F, I). From our example, it is evident that there is a batch effect between the two datasets (Figure 2F, I). Therefore, we applied empirical Bayes batch correction and can see the batch effect has been removed in the density and box-and-whisker plots (Figure 2G, J). Additionally, we can see the dataset is not normalised (Figure 2G, J) and therefore normalisation should be applied during DGEA.

GEOexplorer performs PCA and displays the results in four interactive plots: 1) a scree plot displaying the percentage of the variance in the gene expression dataset captured by each principal component (PC); 2) an interactive individuals plot, reporting for each probe, the value of the PC with the greatest variance (PC1) plotted against the value for the PC with the second greatest variance (PC2); 3) an interactive variables plot reporting for each experimental condition, the PC1 value plotted against the PC2 value; 4) a three dimensional version of this plot (Figure 2K) reporting for each experimental condition, the PC1 value plotted against the PC2 value and the PC with the third greatest variance (PC3). The variables plots are useful for identifying experimental conditions that have closely related probe expression values.

GEOexplorer displays an interactive uniform manifold approximation and projection (UMAP) plot using the *UMAP* R package, available from https://cran.r-project.org/web/packages/umap/index.html. The algorithm initially builds a graph connecting each experimental condition in the dataset. Each experimental condition is given an edge to each of its nearest neighbours (20). This helps to group experimental conditions with their nearest neighbours during the subsequent optimisation step. The number of nearest neighbours used can be updated by the user.

GEOexplorer calculates the covariances/correlations between each of the experimental conditions and displays them in a heatmap (Figure 2H). Like the UMAP and PCA variables plots, the heatmap plot helps to group similar experimental conditions. These groups can then be used in DGEA. From our example, the Heatmap identified 5 closely related samples GSM2836956, GSM2836954, GSM2836946, GSM2836944 and GSM2836935. To validate if they are outliers and should be removed from the analysis, we used the PCA variables plots (Figure 2K) and UMAP plot to see if they cluster using linear and non-linear dimensionality reduction techniques. As they did not cluster in the PCA variables and UMAP plots, and considering the aims of this case study, we did not remove them from the analysis.

### Generating and exploring the results of DGEA

In this case study, the microarray expression dataset post-transformation is used as the basis of DGEA. It, therefore, incorporates the log_2_ transformation and KNN configurations set during data transformation, whereas, if RNA-seq expression data is used in GEOexplorer, pre-transformation is used as the basis of DGEA and therefore does not incorporate log_2_ or counts per million transformations. GEOexplorer will check if the RNA-seq expression dataset does not contain transformed data before DGEA and raises a warning if transformed data are detected.

**Figure 3.**
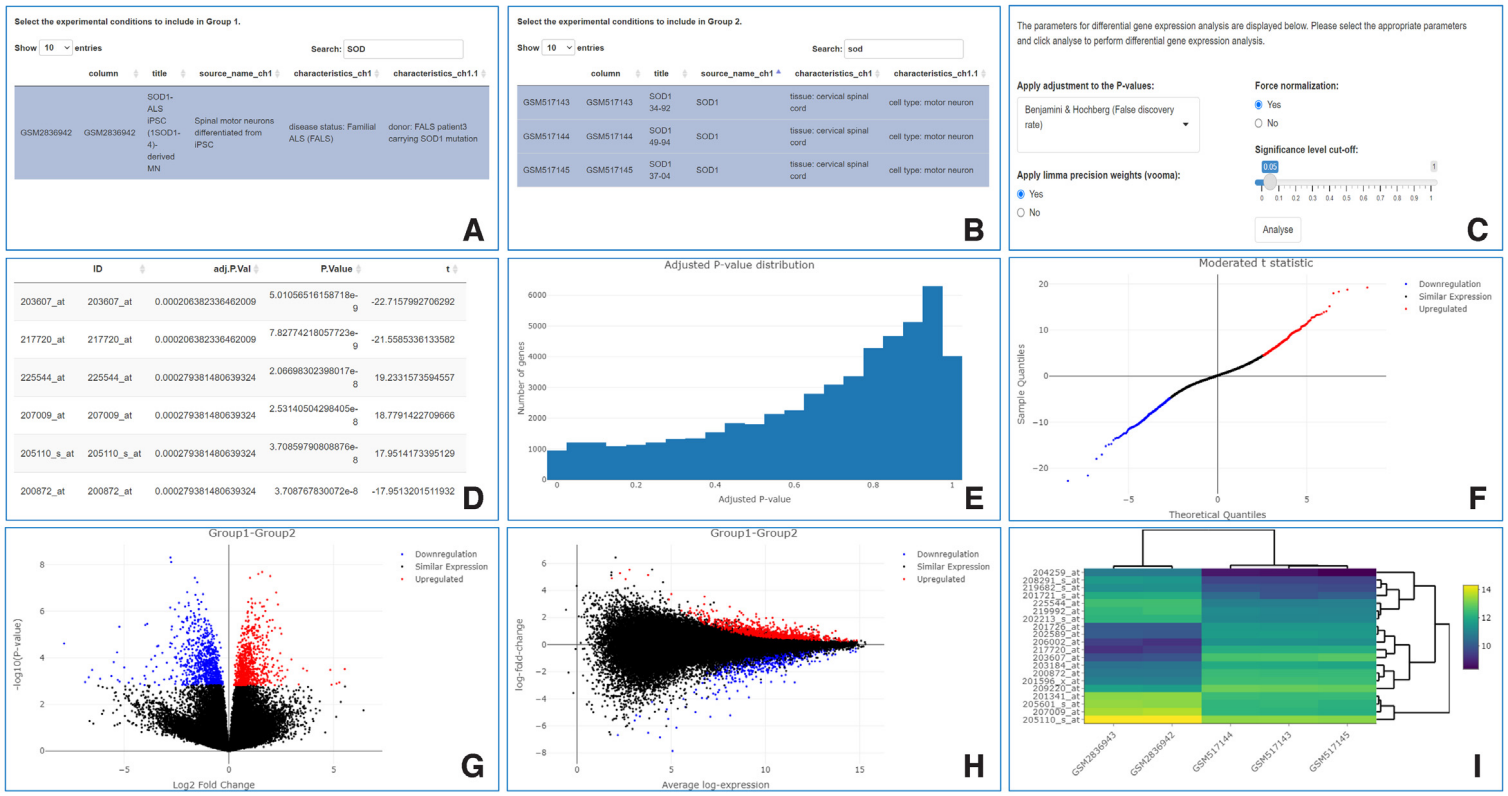
GEOexplorer DGEA analysis settings and DGEA outputs. **(A)** Sample selection for group 1. (**B)** Sample selection for group 2. (**C)** DGEA options. (**D)** Table of the differentially expressed probes. (**E)** Histogram plot of adjusted P-values. (**F)** Quantile-quantile (QQ) plot. (**G)** Volcano plot. (**H)** Mean difference plot. (**I)** Heatmap plot.

To perform DGEA the user must select samples to include in group 1 and group 2 that reflect the experimental conditions to investigate (Figure 3A, B). In our example, we compare the 2 model SOD1 spinal motor neurons, GSM2836942 and GSM2836943, against the 3 post-mortem SOD1 spinal motor neurons, GSM517143, GSM517144, and GSM517145. The probe expression values for the experimental conditions in group 1 will then be compared to the probe expression values for the experimental conditions in group 2. To estimate the variability and perform the statistical tests required to perform DGEA, each group needs to include at least 2 samples.

Several different options can be selected during DGEA (Figure 3C). This includes selecting from the following P-value adjustments; Benjamini & Hochberg (False discovery rate), Benjamini & Yekutieli, Bonferroni, Holm, or none. Whether to use limma precision weights, to force normalisation and the significance level cut off can all be selected. DGEA of RNA-seq datasets should use forced normalisation as RNA-seq raw counts by nature are not normalised. Forcing normalisation ensures that the expression distributions of each sample are similar across the entire experiment and therefore comparable. DGEA of RNA-seq datasets should use limma precision weights as the variance of RNA-seq datasets is not independent of the mean (21). From our example, we used Benjamini & Hochberg (False discovery rate) P-value adjustment and forced normalisation as the data were not normalised (Figure 2G, J), applied limma precision weights as there was a mean-variance trend (Figure 2E) and used a significance level cut off of 0.05.

After selecting the required options, clicking the ‘Analyse’ button triggers DGEA. GEOexplorer uses multiple functions from the *limma* R package (6) to perform DGEA. If the user selected forced normalisation, the expression intensities across the two groups of experimental conditions are normalised and therefore consistent. If the user selects to use limma precision weights, then the mean-variance relationship is estimated, and the observational-level weights are calculated from the mean-variance relationship.

DGEA is done by fitting a linear model to each probe within the dataset. The linear model estimates the fold change in the expression of a probe (or of a gene/transcript in case of an RNA-seq experiment) while considering standard errors by applying empirical Bayes smoothing. Probes (or genes/transcripts in case of an RNA-seq experiment) are subsequently ranked based on their fold change values.

GEOexplorer displays the results of DGEA in several visualisations, including the examples in Figure 3D-G, to help explore the results. These include a table of the differentially expressed probes (or genes/transcripts in case of an RNA-seq experiment), a histogram, a Venn diagram, a QQ plot, a volcano plot, and a mean difference plot.

The table of the differentially expressed probes (or genes/transcripts in case of an RNA-seq experiment) contains the DGEA statistics (Figure 3D) and incorporates the P-value adjustment selected by the user. From our example, we could see there were several differentially expressed probes based on the adjusted P-values < 0.05. The interactive histogram displays the distribution of adjusted P-values across all the probes (or genes/transcripts in case of an RNA-seq experiment) as shown in Figure 3E. This is useful for determining if an appropriate P-value adjustment was selected.

The QQ plot displays the quantiles of the differentially expressed probes (or genes/transcripts in case of an RNA-seq experiment) plotted against the theoretical quantiles of a Student's t distribution (Figure 3F). From our example, we can see the QQ plot broadly follows a straight line which indicates the moderated t-statistics computed during the DGEA follow their theoretically predicted distribution. This indicates the DGEA is not inflated (6).

The volcano plot displays the statistical significance (-log_10_ P-value) versus magnitude of change (log_2_ fold change) for each probe (or genes/transcripts in case of an RNA-seq experiment) (Figure 3G).

The mean difference plot displays the log_2_ fold change against the average log_2_ expression values for each probe (or genes/transcripts in case of an RNA-seq experiment) (Figure 3H).

In the QQ, volcano and mean difference plots, the upregulated probes (or genes/transcripts in case of an RNA-seq experiment) are highlighted in red, and the downregulated ones are highlighted in blue. From our example, we can see there are 1,590 differentially expressed probes in the model SOD1 spinal motor neurons compared to the post-mortem SOD1 spinal motor neurons (Figure 3F-H).

GEOexplorer also displays a heatmap plot containing the expression values, for the differentially expressed probes (or genes/transcripts in case of an RNA-seq experiment) with the lowest adjusted P-values, for each of the experimental conditions analysed in DGEA (Figure 3I). The user can update the number of differentially expressed probes (or genes/transcripts in case of an RNA-seq experiment) to display.

### Generating and exploring the results of GEA

**Figure 4.**
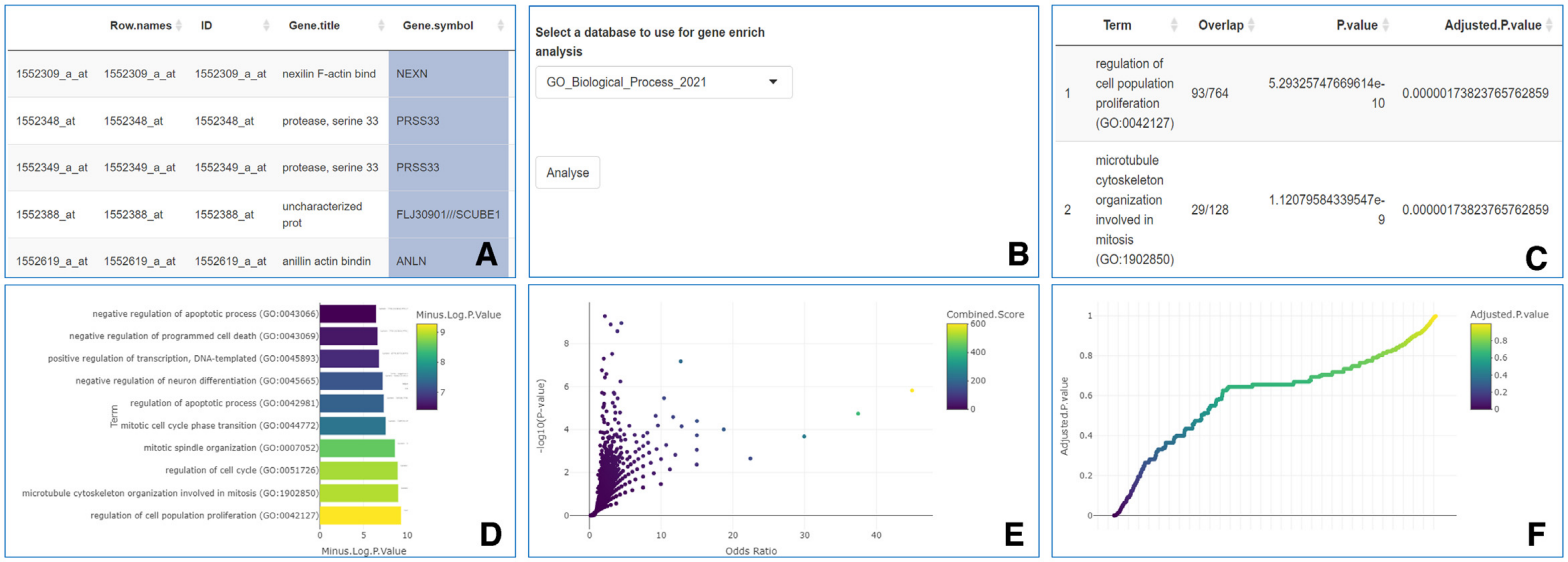
GEOexplorer GEA settings and GEA outputs. **(A)** Selecting the gene symbols. (**B)** GEA options. (**C)** Table of enriched terms. (**D)** Bar chart of the top enriched terms. (**E)** Volcano plot. (**F)** Manhattan plot.

To identify the biological mechanisms and processes underlined by the results of the DGEA, the differentially expressed genes are used for GEA. To perform GEA, the user must select the column containing the gene symbols and fill in any missing gene symbols (Figure 4A). After that, the user can select the Enrichr database they wish to use for GEA (Figure 4B). In our examples, we selected the GO biological processes 2021 database to identify the biological processes enriched by the differentially expressed genes. After clicking the ‘Analyse’ button GEA is triggered. GEOexplorer uses the *enrichR* R package (14–16) to perform GEA.

The enrichment results are returned to the user in the form of a table (Figure 4C), a bar chart of the top enriched terms (Figure 4D), a volcano plot (Figure 4E) and a manhattan plot (Figure 4F). Each of these can display the enrichment results for all the differentially expressed genes, the upregulated genes, or the downregulated genes. The user can select the statistic to display on the x-axis of the bar chart and the y-axis of the manhattan plot. From our example, the GEA highlighted key differences in the cell development and lifecycle of the two groups (Figure 4C, D). Indeed, the top 10 enriched GO terms, according to their adjusted p-value, highlighted the overrepresentation among the differentially expressed genes, of genes involved in cell development and lifecycle pathways such as the ‘regulation of cell population proliferation’, ‘microtubule cytoskeleton organisation involved in mitosis’ and ‘negative regulation of apoptotic process’. This reflects well the process to differentiate the iPSCs into motor neurons in the model SOD1 spinal motor neurons group as well as the post-mortem provenance of the other group.

## DISCUSSION AND CONCLUSION

GEOexplorer has been designed to enable gene expression analysis of user uploaded gene expression data and the datasets available in GEO without the need to be proficient at programming or possess advanced bioinformatics skills. The webserver achieves this by making a comprehensive and standardised, end-to-end gene expression analysis protocol that includes a broad range of tools for EDA, DGEA, GEA and dataset harmonisation as well as the search and retrieval of GEO datasets, available via its easy-to-use graphical user interface (GUI).

The rich selection of analysis and interactive outputs constitutes a significant advantage for uncovering new biological insights from expression datasets. Moreover, GEOexplorer provides a platform to facilitate discoveries in a standardised way, which consequently improves the reproducibility of the analyses. Despite being highly flexible in terms of the characteristics of datasets it can handle, the GEOexplorer protocol requires some manual steps, e.g. the selection of samples for each group in the DGEA, which would be impractical for very large datasets that include thousands of samples. Furthermore, because of the high variability in the format used by RNA-seq GEO datasets, at least at present, GEOexplorer can automatically retrieve and process only approximately a third of the GEO RNA-seq datasets, with the remainder having to be manually downloaded, formatted, and uploaded by the user. This remains a consistent unmet need, particularly considering the current focus of data generation on RNA-seq in preference to older microarray technology. These aspects will be the focus of our future development of the webserver. Finally, for an optimal use of the server, it is beneficial for GEOexplorer's users to have some basic knowledge of gene expression analysis. Several articles can provide users with a general overview of gene expression analysis (1) as well as an in-depth look into microarray (3) and RNA-seq gene expression analysis (4,5).

## DATA AVAILABILITY

The GEOexplorer webserver is available at the following URL https://geoexplorer.rosalind.kcl.ac.uk/. The GEOexplorer package can be downloaded from its Bioconductor page http://bioconductor.org/packages/GEOexplorer/ or the GitHub development page https://github.com/KHP-Informatics/GEOexplorer.

Datasets used in the described example are available from (17-18), GEO Series ID: GSE106382 and GEO Series ID: GSE20589.
